# SNAP23/25 and VAMP2 mediate exocytic event of transferrin receptor-containing recycling vesicles

**DOI:** 10.1242/bio.012146

**Published:** 2015-06-19

**Authors:** Keiji Kubo, Minako Kobayashi, Shohei Nozaki, Chikako Yagi, Kiyotaka Hatsuzawa, Yohei Katoh, Hye-Won Shin, Senye Takahashi, Kazuhisa Nakayama

**Affiliations:** 1 Graduate School of Pharmaceutical Sciences, Kyoto University, Sakyo-ku, Kyoto 606-8501, Japan; 2 Division of Molecular Biology, Tottori University School of Life Science, Yonago, Tottori 683-8503, Japan

**Keywords:** SNAP23, SNAP25, VAMP2, Exocyst, Recycling endosome, Transferrin receptor, Exocytosis

## Abstract

We recently showed that Rab11 is involved not only in formation of recycling vesicles containing the transferrin (Tfn)–transferrin receptor (TfnR) complex at perinuclear recycling endosomes but also in tethering of recycling vesicles to the plasma membrane (PM) in concert with the exocyst tethering complex. We here aimed at identifying SNARE proteins responsible for fusion of Tfn–TfnR-containing recycling vesicles with the PM, downstream of the exocyst. We showed that exocyst subunits, Sec6 and Sec8, can interact with SNAP23 and SNAP25, both of which are PM-localizing Q_bc_-SNAREs, and that depletion of SNAP23 and/or SNAP25 in HeLa cells suppresses fusion of Tfn–TfnR-containing vesicles with the PM, leading to accumulation of the vesicles at the cell periphery. We also found that VAMP2, an R-SNARE, is colocalized with endocytosed Tfn on punctate endosomal structures, and that its depletion in HeLa cells suppresses recycling vesicle exocytosis. These observations indicate that fusion of recycling vesicles with the PM downstream of the exocyst is mediated by SNAP23/25 and VAMP2, and provide novel insight into non-neuronal roles of VAMP2 and SNAP25.

## INTRODUCTION

Cells internalize extracellular materials, plasma membrane (PM) proteins and their ligands by endocytosis. Some of endocytosed proteins are recycled back to the cell surface for reuse, whereas others are destined for degradation in lysosomes. Transferrin receptor (TfnR) and its ligand, transferrin (Tfn), have been used to trace the endocytic and recycling processes in a number of studies (Maxfield and McGraw, 2004; Mayle et al., 2012). TfnR binds Fe^3+^-loaded Tfn at the cell surface, and is internalized via clathrin-coated vesicles and delivered to early/sorting endosomes. The Tfn−TfnR complex is returned to the PM either directly from early endosomes (rapid recycling) or indirectly via recycling endosomes (REs) (slow recycling) after release of loaded Fe^3+^ (Grant and Donaldson, 2009; Maxfield and McGraw, 2004; Mayle et al., 2012). REs, whose subcellular localization varies among cell types, are often located near the nucleus or centrosome and consequently referred to as perinuclear or pericentrosomal REs.

Formation of recycling vesicles containing the Tfn–TfnR complex at REs is regulated by a Rab family small GTPase, Rab11, since expression of a dominant-negative Rab11a mutant, Rab11a(S25N), inhibited formation of recycling vesicles and consequently induced tubulation of perinuclear compartments containing endocytosed Tfn and TfnR (Hölttä-Vuori et al., 2002; Takahashi et al., 2012; Wilcke et al., 2000). We recently showed that, in addition to its role at perinuclear REs, Rab11 regulates tethering/fusion of Tfn–TfnR-containing recycling vesicles with the PM in concert with the exocyst tethering complex (Takahashi et al., 2012); siRNA-mediated depletion of Rab11a and Rab11b caused accumulation of endocytosed Tfn and TfnR beneath the PM and extremely decreased the frequency of exocytic events of TfnR-EGFP-containing vesicles detected by total internal reflection fluorescence microscopy (TIRFM). Furthermore, we showed that depletion of subunits of the exocyst tethering complex led to essentially the same phenotype as that observed in Rab11-depleted cells; in exocyst-depleted cells, endocytosed Tfn and TfnR were accumulated beneath the PM and the exocytic frequency of TfnR-containing vesicles was extremely reduced (Takahashi et al., 2012). Since GTP-bound Rab11 interacts with an exocyst subunit (Wu et al., 2005; Zhang et al., 2004) (also see Fig. 8), we concluded that Rab11, in concert with the exocyst, is involved in tethering of Tfn–TfnR-containing recycling vesicles with the PM (Takahashi et al., 2012).

After tethering of vesicles with the target membrane, a vesicle SNARE (R-SNARE) and target SNAREs (Q-SNAREs) form a *trans*-SNARE complex to mediate the final membrane fusion step (Hong and Lev, 2014). Our next question is, therefore, which SNARE proteins mediate fusion of recycling vesicles with the PM downstream of the exocyst. Previous *in vitro* studies showed that a yeast exocyst subunit, Sec6p, can interact with Sec9p (Morgera et al., 2012; Sivaram et al., 2005), which is a target-SNARE/Q_bc_-SNARE homologous to mammalian SNAP23 and SNAP25. On the other hand, Tfn recycling in mammalian cells was reported to be sensitive to botulinum neurotoxin E (Leung et al., 1998) and tetanus toxin (TeTX) (Galli et al., 1994), both of which are metalloproteinases cleaving SNAP23 and SNAP25, and VAMP2 and VAMP3 (vesicle-SNAREs/R-SNAREs), respectively.

In this study, we first showed that SNAP23 and SNAP25 are able to interact with exocyst subunits, Sec6 and Sec8, respectively, and involved in fusion of Tfn–TfnR-containing recycling vesicles with the PM. By comparing subcellular localization of R-SNAREs and endocytosed Tfn, we found that a population of VAMP2 is colocalized with endocytosed Tfn on punctate endosomal structures and showed that siRNA-mediated knockdown of VAMP2 specifically suppresses exocytosis of recycling vesicles containing Tfn and TfnR.

## RESULTS

### Interaction of Sec6 and Sec8 with SNAP23 and SNAP25

**Fig. 1.**
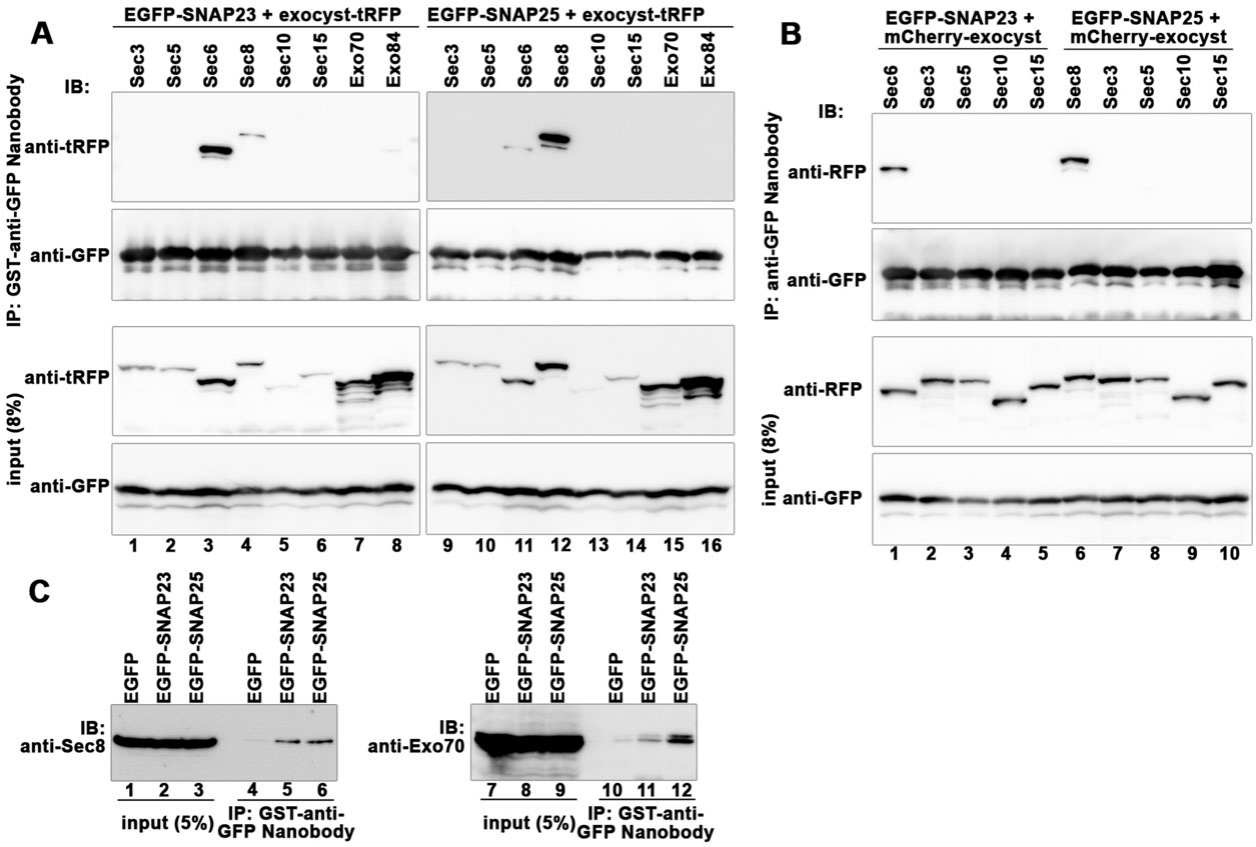
**Interaction of exocyst subunits with SNAP23 and SNAP25.** (A) HEK293T cells were cotransfected with expression vectors for either EGFP-SNAP23 (lanes 1-8) or EGFP-SNAP25 (lanes 9-16) and C-terminally tRFP-tagged exocyst subunit indicated, except for Sec6, which was N-terminally tRFP-tagged (lanes 3 and 11). Lysates prepared from the cotransfected cells were subjected to immunoprecipitation (IP) using GST-tagged anti-GFP Nanobody prebound to glutathione–Sepharose 4B beads, followed by immunoblot (IB) analysis using anti-tRFP (upper panels) or anti-GFP (lower panels) antibody. (B) HEK293T cells were cotransfected with expression vectors for either EGFP-SNAP23 (lanes 1-5) or EGFP-SNAP25 (lanes 6-10) and an exocyst subunit indicated (lanes 1-5 and lanes 6-10) with either an N-terminal (lanes 1, 4, 5, 9 and 10) or C-terminal (lanes 2, 3 and 6-8) mCherry-tag. Lysates prepared from the cotransfected cells were subjected to immunoprecipitation (IP) using GST–anti-GFP Nanobody prebound to glutathione-Sepharose 4B beads and following immunoblot (IB) analysis using anti-RFP (upper panel) or anti-GFP (lower panel) antibody. (C) HEK293T cells were transfected with an expression vector for EGFP-SNAP23 or -SNAP25, and lysates prepared from the transfected cells were subjected to immunoprecipitation using GST–anti-GFP Nanobody prebound to glutathione–Sepharose 4B beads, followed by immunoblot analysis using antibody against Sec8 (lanes 1-6) or Exo70 (lanes 7-12).

In view of our previous data showing that, downstream of Rab11, the exocyst is involved in the exocytic event of Tfn–TfnR-containing recycling vesicles (Takahashi et al., 2012), we asked which SNAREs are responsible for recycling of Tfn–TfnR downstream of the exocyst. Previous studies in yeasts showed that an exocyst subunit, Sec6p, interacts with Sec9p *in vitro* (Morgera et al., 2012; Sivaram et al., 2005); Sec9p is homologous to mammalian PM-localizing Q_bc_-SNAREs, SNAP23 and SNAP25. Therefore, we first addressed whether the exocyst-SNARE interaction is conserved in mammalian cells. To this end, we expressed EGFP-tagged SNAP23 or SNAP25 in combination with any one of the exocyst subunits fused to tagRFP (tRFP) in HEK293T cells, and lysates prepared from these cells were subjected to immunoprecipitation with GST–anti-GFP Nanobody and subsequent immunoblot analysis. In line with the yeast Sec6p–Sec9p interaction, tRFP-Sec6 was co-immunoprecipitated with EGFP-SNAP23 (Fig. 1A, lane 3). Sec8-tRFP was also co-immunoprecipitated with EGFP-SNAP23 (lane 4), although much less efficiently than tRFP-Sec6. On the other hand, EGFP-SNAP25 efficiently co-precipitated Sec8-tRFP (lane 12) and much less efficiently tRFP-Sec6 (lane 11). These results suggest that SNAP23 interacts directly with Sec6 and indirectly with Sec8 through Sec6, while SNAP25 interacts directly with Sec8 and indirectly with Sec6 through Sec8, since previous studies showed a Sce6–Sec8 interaction (Munson and Novick, 2006).

Since the cellular expression levels of tRFP-tagged Sec3, Sec5, Sec10 and Sec15 were relatively low compared with other exocyst subunits, we also constructed vectors for mCherry-tagged versions of these exocyst subunits, whose expression was under the control of a strong promoter (CAG; chicken actin gene), and coexpressed the mCherry-tagged exocyst subunit with EGFP-SNAP23 or -SNAP25 in HEK293T cells. As shown in Fig. 1B, although the expression levels of Sec3, Sec5, Sec10 and Sec15 were indeed improved, we failed to show interactions of Sec3, Sec5, Sec10 and Sec15 with SNAP23 and SNAP25. These results show for the first time that mammalian SNAP23 and SNAP25 interact with specific subunits of the exocyst, Sec6 and Sec8, respectively, and suggest that these Q_bc_-SNAREs can be involved in exocytic events downstream of the exocyst.

To address whether SNAP23 and SNAP25 interact with the assembled exocyst complex, we processed lysates of cells expressing EGFP-SNAP23 or -SNAP25 for immunoprecipitation with GST–anti-GFP Nanobody and subsequent immunoblot analysis with available antibodies against exocyst subunits. As shown in Fig. 1C, endogenous Sec8 was co-immunoprecipitated with both EGFP-SNAP23 and -SNAP25 (lanes 5 and 6). Given that EGFP-SNAP23 interacted with Sec8-tRFP much less efficiently than with Sec6-tRFP (Fig. 1A, lanes 3 and 4) while EGFP-SNAP25 interacted with Sec6-tRFP much less efficiently than with Sec8-tRFP (Fig. 1A, lanes 11 and 12), the result suggest that SNAP23 interacted with Sec8 indirectly through Sec6. Furthermore, endogenous Exo70 was co-immunoprecipitated with both EGFP-SNAP23 and -SNAP25 (Fig. 1C, lanes 11 and 12). Taken together with the data shown in Fig. 1A and B, it is likely that SNAP23 and SNAP25 can interact with at least some fractions of the assembled exocyst complex through Sec6 and Sec8, respectively.

### Participation of SNAP23 and SNAP25 in fusion of Tfn–TfnR-containing recycling vesicles with the PM

To examine whether SNAP23 and/or SNAP25 are involved in recycling of Tfn–TfnR, we treated HeLa cells with siRNAs for either SNAP23 or SNAP25 alone or with their combination, and examined Tfn–TfnR recycling in these cells. Specific and efficient depletion of SNAP23 or SNAP25 by the siRNA treatment was confirmed by immunoblot analysis (supplementary material Fig. S1). In addition, the immunoblot analysis unequivocally showed that SNAP25 as well as SNAP23 is expressed in HeLa cells (supplementary material Fig. S1), although previous studies reported that SNAP25 was expressed predominantly in neuronal tissues while SNAP23 was expressed ubiquitously (Ravichandran et al., 1996; Scott et al., 2003).

**Fig. 2.**
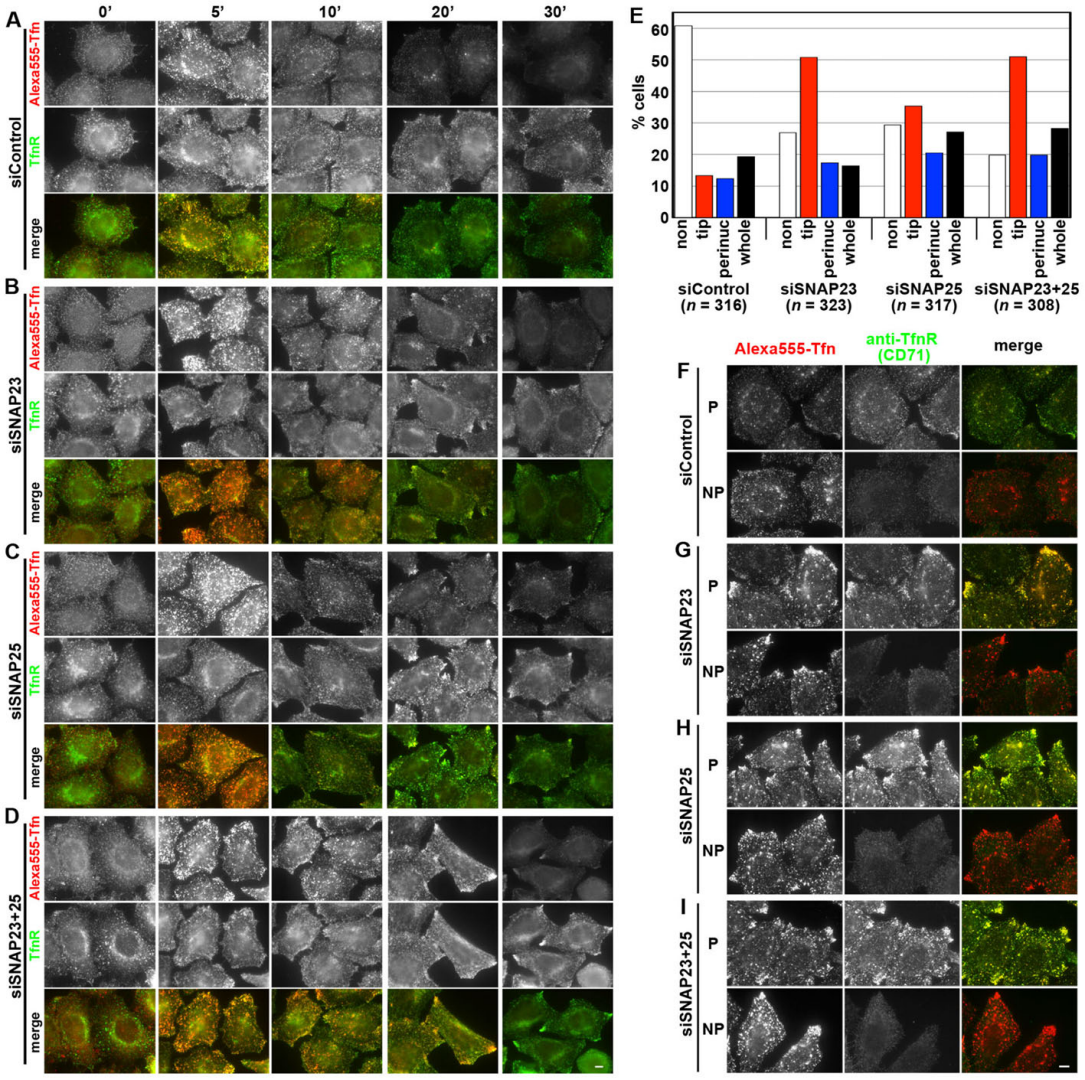
**Accumulation of recycled Tfn and TfnR beneath the PM in cells knocked down of SNAP23 and/or SNAP25.** HeLa cells transfected with control siRNAs (A; siRNAs for LacZ) or siRNAs for SNAP23 (B) or SNAP25 (C), or for both SNAP23 and SNAP25 (D) were serum-starved for 3 h and incubated with AlexaFluor 555-conjugated Tfn at 4°C for 50 min. After washing out excess fluorescent Tfn, the cells were incubated in serum-containing medium at 37°C for the indicated time periods to allow endocytosis and recycling, and processed for immunostaining with anti-TfnR antibody (H68.4) after fixation and permeabilization. Representative images from one of three sets of experiments are shown. In particular, in cells knocked down of both SNAP23 and SNAP25, both endocytosed Tfn and TfnR were significantly accumulated in the tip regions, compared with control cells. (E) Cells incubated at 37°C for 20 min in a set of experiments shown in A-D were classified into those with AlexaFluor 555-Tfn signals below the detection limit (non, white), primarily around the cellular tips (tip, red), primarily in the perinuclear region (perinuc, blue), and distributed throughout the cell (whole, black). The percentages of the counted cells were expressed as bar graphs. (F-I) Cells incubated at 37°C for 20 min as in A-D were fixed, and, immediately (NP) or after permeabilization (P), processed for immunostaining with anti-TfnR antibody (CD71), which recognizes the exoplasmic region of TfnR. Scale bars=10 μm.

In HeLa cells treated with control siRNAs, AlexaFluor 555-conjugated Tfn bound to the cell surface at 4°C was internalized into the cells within 5 min incubation at 37°C, and subsequently recycled and secreted into the medium; most of endocytosed fluorescent Tfn disappeared from the cells by 20 min at 37°C (Fig. 2A). In contrast, cells knocked down of SNAP23 exhibited a delay in the recycling process of fluorescent Tfn. Although there was a tendency of reduced accumulation of endocytosed Tfn in the perinuclear region after 5 min incubation at 37°C, the most prominent feature of the SNAP23-knockdown cells was accumulation of endocytosed Tfn and TfnR at the cell periphery, in particular, around the cellular tips after 20 or 30 min incubation at 37°C (Fig. 2B); this phenotype resembled that observed in cells knocked down of Rab11 or an exocyst subunit as described previously (Takahashi et al., 2012). Cells knocked down of SNAP25 also showed a tendency of delayed recycling of Tfn–TfnR (Fig. 2C). Accumulation of Tfn and TfnR at the cell periphery in cells subjected to simultaneous knockdown of SNAP23 and SNAP25 were more prominent than those subjected to single knockdown of SNAP23 or SNAP25 (Fig. 2D).

When morphologically examined, cells knocked down of SNAP23 and/or SNAP25 exhibited a tendency of delayed Tfn recycling (Fig. 2E). After 20 min incubation at 37°C, AlexaFluor 555-Tfn signals were below the detection limit in ∼60% of control cells, while only in ∼20-30% of cells knocked down of SNAP23 or SNAP25. On the other hand, ∼50 and ∼35% of SNAP23- and SNAP25-knckdown cells, respectively, had Tfn signals in the cellular tip regions, while only ∼10% of control cells had Tfn signals around the cellular tips.

We then asked whether Tfn and TfnR accumulated at the cell periphery are on or beneath the PM. To this end, siRNA-treated cells were incubated with AlexaFluor 555-Tfn at 37°C for 20 min and immunostained with anti-TfnR antibody (CD71), which recognizes the exoplasmic/lumenal epitope of TfnR, under either permeabilized or non-permeabilized conditions. As shown in Fig. 2F-I, TfnR detected in the tip regions under permeabilized conditions (P) was not recognized by the CD71 anti-TfnR antibody under non-permeabilized conditions (NP), indicating accumulation of TfnR beneath the PM. Thus, the significant accumulation of Tfn and TfnR beneath the PM in cells knocked down of SNAP23 and/or SNAP25 suggests that these Q_bc_-SNAREs are involved in fusion of recycling vesicles containing Tfn–TfnR with the PM.

**Fig. 3.**
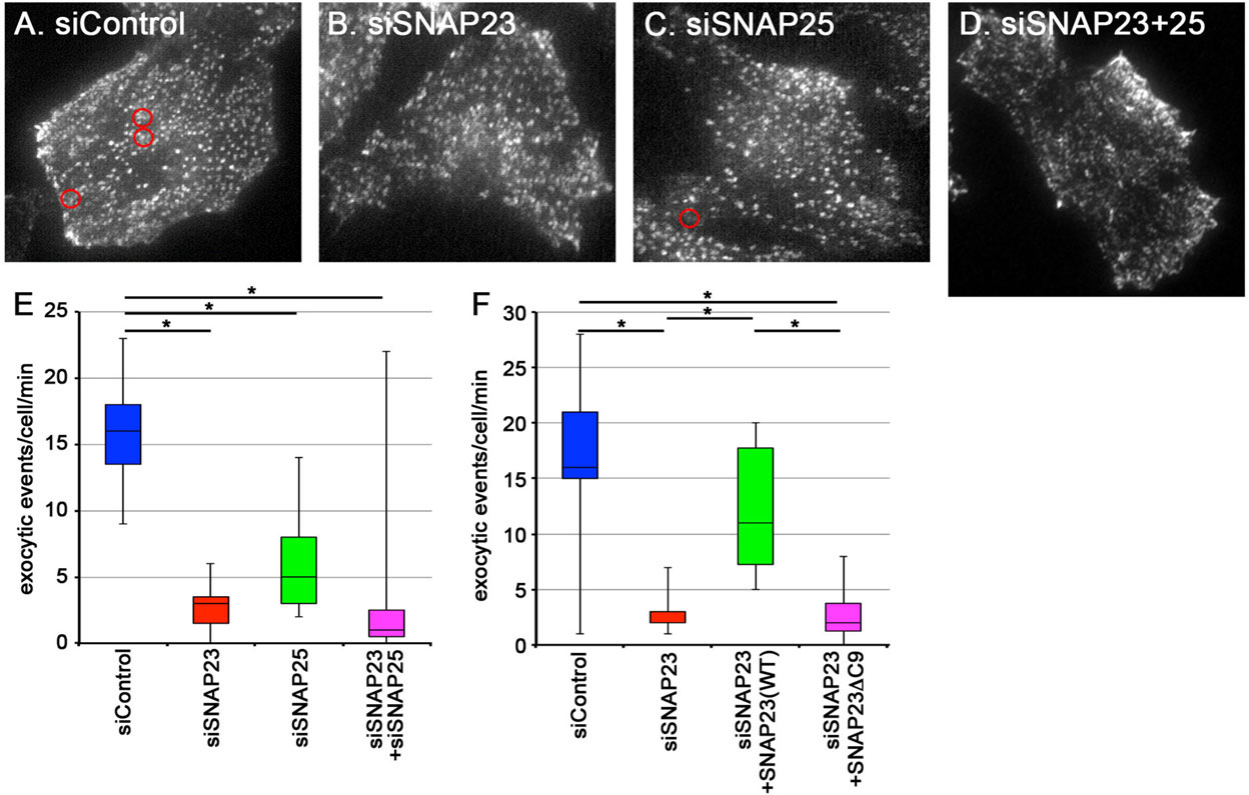
**Knockdown of SNAP23 and/or SNAP25 inhibits exocytic events of TfnR-EGFP-containing vesicles.** (A-E) HeLa cells stably expressing TfnR-EGFP were transfected with control siRNAs (A), or siRNAs for SNAP23 (B) or SNAP25 (C), or for both SNAP23 and SNAP25 (D), and subjected to TIRFM to reveal fusion of TfnR-EGFP-containing recycling vesicles with the PM. Representative frames from the corresponding supplementary material Videos S1-S4 are shown. Positions of typical exocytic events are circled in red. (E) Exocytic events detected per minute in single cells were counted in each case (*n*=15) and expressed as box and whisker plots with the median values. (F) HeLa cells stably expressing TfnR-EGFP were transfected with control (*n*=10) or SNAP23 siRNAs (*n*=13), or SNAP23 siRNAs along with an expression vector for mCherry-tagged SNAP23(WT) (*n*=10) or SNAP23ΔC9 (*n*=10), and subjected to TIRFM. Exocytic events detected per minute in single cells were counted in each case and expressed as box and whisker plots with the median values. *P<0.05.

The above morphological analysis of cells knocked down of SNAP23 and/or SNAP25 suggested involvement of these Q_bc_-SNAREs in fusion of recycling vesicles with the PM, but failed to show a statistically significant difference between the control and SNAP23/25-knockdown cells due to variability among sets of experiments, a limitation associated with this type of morphological analysis. To circumvent this problem, we performed TIRFM analysis of HeLa cells stably expressing TfnR-EGFP (Takahashi et al., 2012; Takatsu et al., 2013) not only to more directly show participation of these Q_bc_-SNAREs in fusion of recycling vesicles with the PM, but also to quantitatively evaluate effects of the Q_bc_-SNARE knockdown on the fusion. In control cells, exocytic events of vesicles containing TfnR-EGFP were often observed, with an average frequency of ∼16 exocytic events/cell/min (Fig. 3A,E, supplementary material Video S1). The exocytic frequency of TfnR-EGFP-containing vesicles was extremely decreased in cells treated with SNAP23 siRNAs (∼3 exocytic events/cell/min) (Fig. 3B,E, supplementary material Video S2) and moderately decreased in cells treated with SNAP25 siRNAs (∼5 exocytic events/cell/min) (Fig. 3C,E, supplementary material Video S3). Simultaneous knockdown of SNAP23 and SNAP25 exhibited a tendency to further decrease the exocytic frequency (∼2 exocytic events/cell/min) (Fig. 3D,E, supplementary material Video S4). As shown in Fig. 3F, the decrease in the exocytic frequency in SNAP23-knockdown cells was significantly recovered by exogenous expression of mCherry-SNAP23(WT), but not by expression of mCherry-SNAP23ΔC9, which mimics an inactive product of SNAP25 subjected to proteolytic cleavage by botulinum neurotoxin A (Huang et al., 2001; Kean et al., 2009). These observations together indicate that SNAP23 is the primary Q_bc_-SNARE involved in fusion of Tfn–TfnR-containing recycling vesicles with the PM and SNAP25 may be redundantly involved in the fusion process.

### Extensive colocalization of VAMP2, VAMP3 and VAMP8 with Tfn–TfnR on punctate endosomal structures

**Fig. 4.**
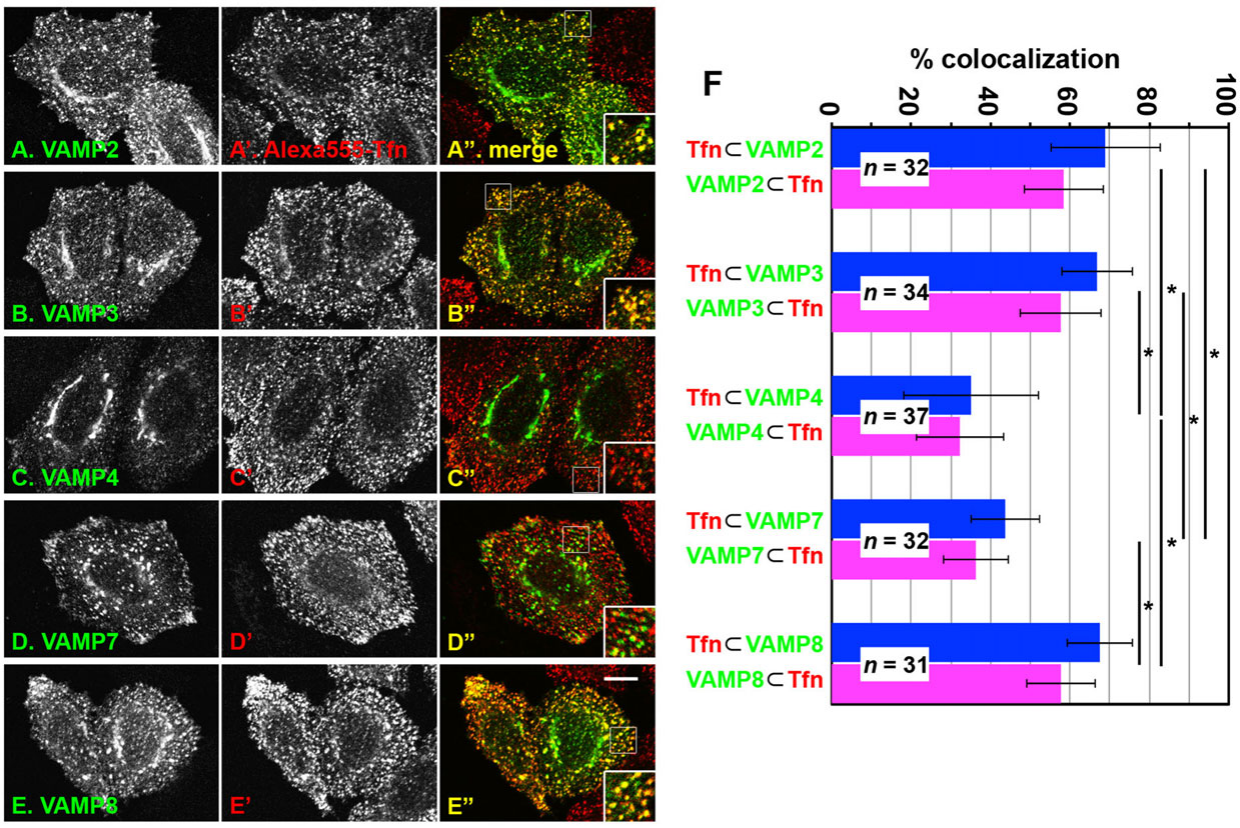
**Comparison of subcellular localization of R-SNAREs with that of endocytosed Tfn.** HeLa cells were transfected with an expression vector for HA-tagged VAMP2 (A-A″), VAMP3 (B-B″), VAMP4 (C-C″), VAMP7 (D-D″) or VAMP8 (E-E″), serum-starved for 3 h, and incubated with AlexaFluor 555-Tfn for 30 min at 37°C (A′-E′). After washing out fluorescent Tfn, the cells were processed for immunostaining with anti-HA antibody (A-E). Images were acquired using a confocal laser-scanning microscope. Bar=10 μm. (F) Colocalization of each VAMP with endocytosed Tfn (blue) or that of endocytosed Tfn with each VAMP (magenta) were estimated using a Measure Colocalization program of MetaMorph (Molecular Devices) and expressed as bar graphs. The values are mean percentages±s.d. of the indicated numbers of cells. *P<0.05.

We then set out to identify R-SNARE(s) involved in the exocytic event of recycling vesicles containing Tfn–TfnR. To this end, we compared subcellular localization of R-SNAREs (VAMP2, VAMP3, VAMP4, VAMP7 and VAMP8), all of which have been reported to localize to endosomal compartments (Hong, 2005), with that of endocytosed AlexaFluor 555-Tfn. Since none of available antibodies against these R-SNAREs we examined, except for anti-VAMP8, worked well to detect endogenous proteins by immunofluorescence analysis, we transfected an expression vector for HA-tagged R-SNAREs into HeLa cells and compared subcellular localization of the expressed protein with that of endocytosed Tfn (Fig. 4). Among these R-SNAREs examined, VAMP2 (Fig. 4A-A″), VAMP3 (Fig. 4B-B″) and VAMP8 (Fig. 4E-E″) exhibited significant colocalization with endocytosed Tfn on punctate endosomal structures. By contrast, VAMP4 (Fig. 4C-C″) and VAMP7 (Fig. 4D-D″), which were reported to be localized mainly on the *trans*-Golgi network and late endosomes, respectively (Hong, 2005), showed less significant colocalization with endocytosed Tfn on punctate structures. Analysis of the acquired images using MetaMorph indicated that VAMP2, VAMP3 and VAMP8 colocalized with endocytosed Tfn more extensively than VAMP4 and VAMP7 (Fig. 4F). Taken into account that VAMP2, VAMP3 and VAMP8 were reported to be localized to early endosomes and REs (Hong, 2005), these observations suggest that these R-SNAREs can function along the endocytic and recycling pathways of Tfn–TfnR.

### VAMP2 is the primary R-SNARE responsible for exocytosis of recycling vesicles containing Tfn–TfnR

**Fig. 5.**
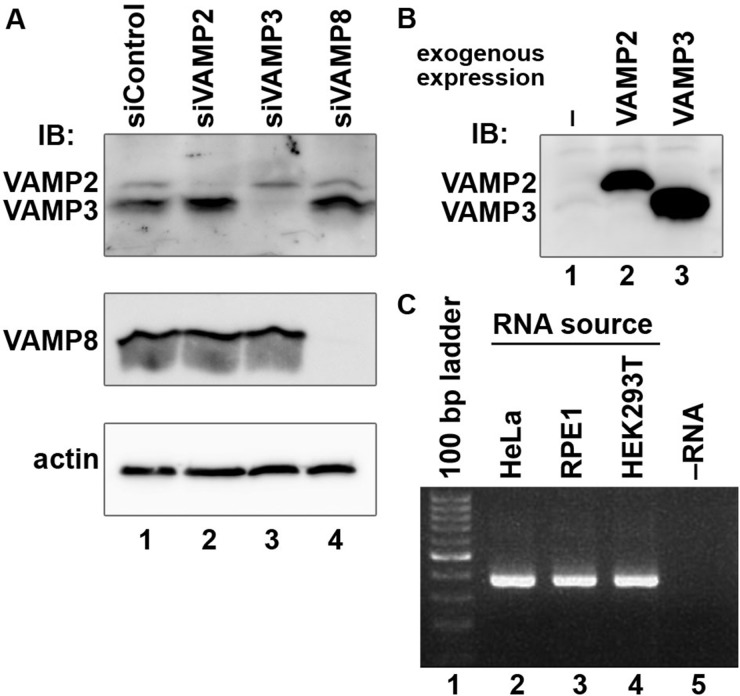
**Confirmation of knockdown of VAMP2, VAMP3 or VAMP8, and expression of VAMP2 in non-neuronal cell lines.** (A) HeLa cells were transfected with control siRNAs (lane 1), or siRNAs for VAMP2 (lane 2), VAMP3 (lane 3) or VAMP8 (lane 4), and lysates prepared from the cells were subjected to immunoblot analysis using antibody that recognizes VAMP2 and VAMP3 (top panel), VAMP8 (middle panel) or actin (bottom panel). (B) HeLa cells transfected with an expression vector for VAMP2 (lane 2) or VAMP3 (lane 3), and lysates from the transfected cells were subjected to immunoblot analysis using the same antibody as that used in A, top panel. (C) Total RNAs isolated from HeLa (lane 2), hTERT-RPE1 (lane 3) or HEK293T (lane 4) cells were subjected to RT-PCR analysis for the VAMP2 transcript using a SuperScript III One-Step RT-PCR System with Platinum Taq DNA polymerase and a primer set used for isolation of its cDNA covering the entire coding region (see supplementary material Table S1); 40 cycles of denature at 94°C for 15 s, annealing at 55°C for 30 s and extension at 68°C for 45 s. The expected size of the amplified cDNA fragment is 373 bp.

On the basis of the colocalization data, we explored possible involvement of VAMP2, VAMP3 or VAMP8 in Tfn–TfnR recycling by siRNA-mediated knockdown of these proteins. Specific and efficient depletion of VAMP proteins in corresponding siRNA-treated HeLa cells was confirmed by immunoblot analysis. In Fig. 5A, middle panel, specific depletion of VAMP8 in HeLa cells was confirmed using anti-VAMP8 antibody. On the other hand, as shown in Fig. 5A, top panel, treatment of HeLa cells with siRNAs for VAMP2 and VAMP3 specifically abolished upper and lower bands, respectively, detected using antibody that recognizes both VAMP2 and VAMP3 (Fig. 5B). The immunoblot data also indicates that both VAMP2 and VAMP3 are expressed in HeLa cells, although the relative expression levels of VAMP2 and VAMP3 could not precisely be determined by the immunoblot data. Previously, VAMP2 was reported to be predominantly expressed in neuronal tissues (Elferink et al., 1989), although it was reported that all but one (VAMP1) VAMPs are expressed in 3T3-L1 adipocytes (Sadler et al., 2015; Williams and Pessin, 2008). We confirmed the presence of the VAMP2 transcript in non-neuronal human cell lines, including HeLa, by RT-PCR (Fig. 5C); it is unlikely that the amplified DNA band was derived from potentially contaminating genomic DNA, because we used a primer set covering the entire coding sequence of the VAMP2 mRNA (supplementary material Table S1) and the coding sequence in the human VAMP2 gene is separated by four introns (Archer et al., 1990). These data suggest that VAMP2 can also function in non-neuronal cells.

**Fig. 6.**
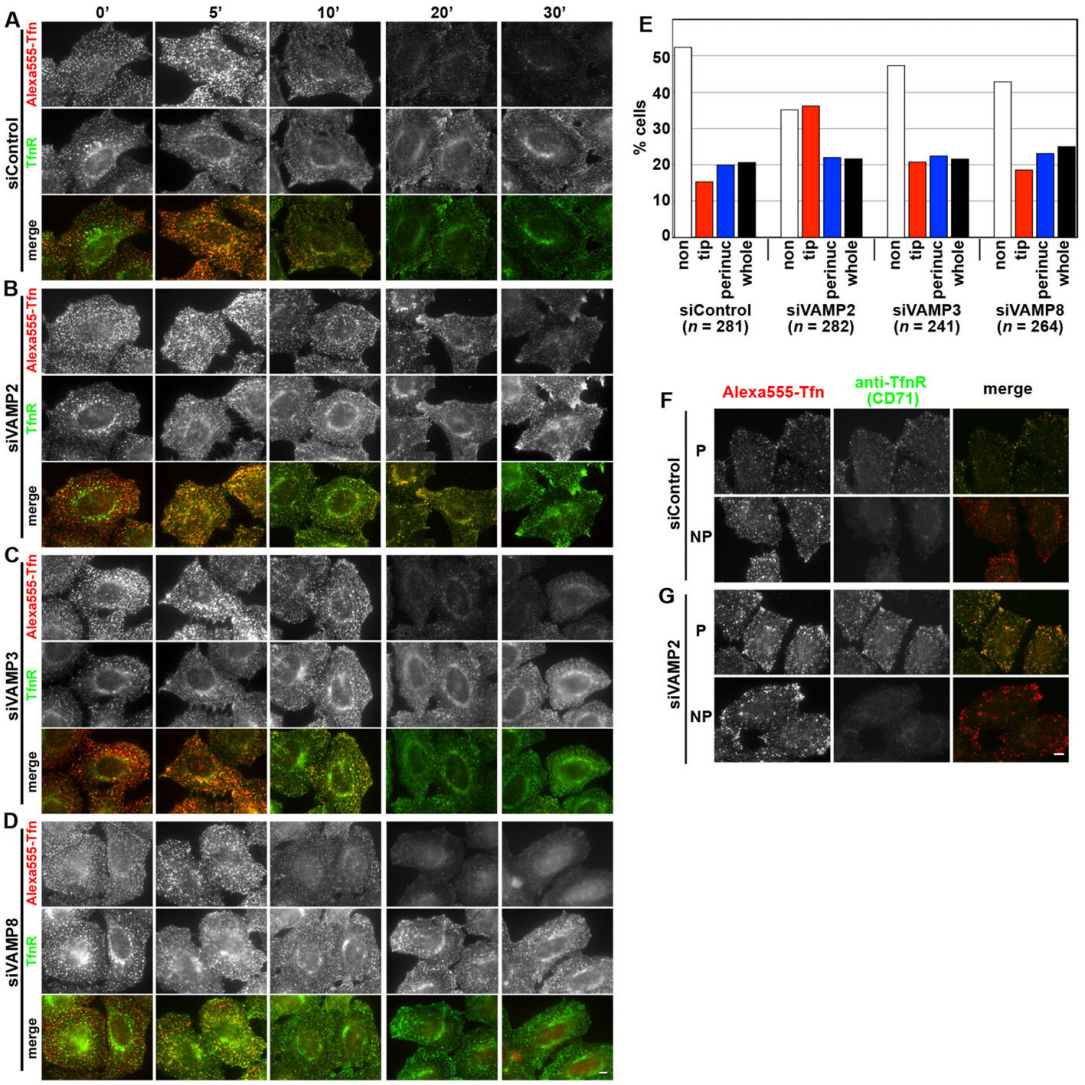
**Accumulation of recycled Tfn and TfnR beneath the PM in VAMP2-knockdown cells.** HeLa cells transfected with control siRNAs (A; siRNAs for LacZ) or siRNAs for VAMP2 (B), VAMP3 (C) or VAMP8 (D) were processed as described in the legend for Fig. 2A-D, and immunostained for TfnR. Representative images from one of three sets of experiments are shown. In the VAMP2-depleted cells, both endocytosed Tfn and TfnR were significantly accumulated in the tip regions, compared with the control cells. (E) Cells incubated at 37°C for 20 min in a set of experiments shown in A-D were classified as described in the legend for Fig. 2E, and the percentages of the counted cells were expressed as bar graphs. (F) and (G), cells incubated at 37°C for 20 min as in A and B were processed as described in the legend for Fig. 2F-I. Scale bars=10 μm.

Compared with control siRNA-treated HeLa cells (Fig. 6A), cells treated with siRNAs for VAMP2 (Fig. 6B) exhibited a retardation in Tfn recycling; the VAMP2-knockdown cells retained endocytosed fluorescent Tfn for longer time than the control cells and showed accumulation of Tfn and TfnR around the cellular tips (also see Fig. 6E), and they also exhibited reduced accumulation of endocytosed Tfn in the perinuclear region. By contrast, treatment of cells with siRNAs for VAMP3 or VAMP8 did not appear to cause a delay in Tfn recycling (Fig. 6C and D, respectively; also see Fig. 6E), compared with control cells. The VAMP3 result was somewhat unexpected, since an early study reported that TeTx impaired Tfn release from permeabilized CHO cells, which correlated with toxin-mediated cleavage of VAMP3, while VAMP2 was below the detection limit in these cells by immunoblot analysis (Galli et al., 1994); both VAMP2 and VAMP3 are substrates for the TeTx metalloproteinase (McMahon et al., 1993; Yamasaki et al., 1994) (see Discussion).

As described for cells knocked down of SNAP23/25 (Fig. 2F-I), TfnR found around the cellular tips in VAMP2-knockdown cells was not detected by the CD71 anti-TfnR antibody under non-permeabilized conditions (Fig. 6F,G), indicating accumulation of TfnR beneath the PM.

**Fig. 7.**
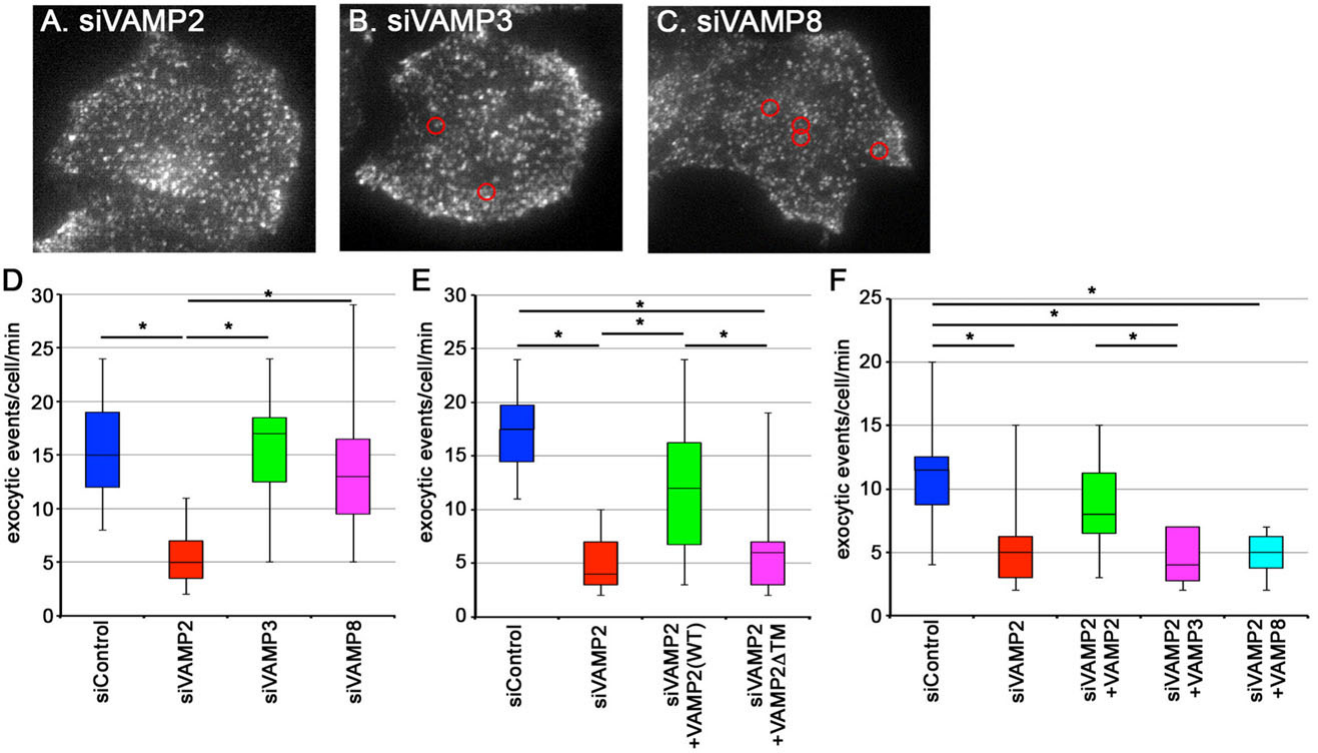
**Knockdown of VAMP2 inhibits exocytic events of TfnR-EGFP-containing vesicles.** (A-D) HeLa cells stably expressing TfnR-EGFP were transfected with control siRNA (not shown), or siRNAs for VAMP2 (A), VAMP3 (B) or VAMP8 (C), and subjected to TIRFM to reveal fusion of TfnR-EGFP-containing recycling vesicles with the PM. Representative frames from the corresponding supplementary material Videos S5-S7 are shown in A-C, respectively. Positions of typical exocytic events are circled in red. (D) Exocytic events detected per minute in single cells were counted in each case (*n*=15), and expressed as box and whisker plots with the median values. (E) HeLa cells stably expressing TfnR-EGFP were transfected with control (*n*=14) or VAMP2 siRNAs (*n*=13), or VAMP2 siRNAs along with an expression vector for mCherry-tagged VAMP2(WT) (*n*=16) or VAMP2ΔTM (*n*=15), and subjected to TIRFM. Exocytic events detected per minute in single cells were counted in each case and expressed as box and whisker plots with the median values. (F) HeLa cells stably expressing TfnR-EGFP were transfected with control siRNAs or VAMP2 siRNAs, or VAMP2 siRNAs along with an expression vector for mCherry-tagged VAMP2, VAMP3, or VAMP8, and subjected to TIRFM. Exocytic events detected per minute in single cells were counted in each case (*n*=12), and expressed as box and whisker plots. *P<0.05.

To evaluate the involvement of VAMP2 and/or VAMP3 in fusion of recycling vesicles with the PM, we next treated HeLa cells stably expressing TfnR-EGFP with VAMP siRNAs and observed the exocytic events of TfnR-EGFP-containing vesicles by TIRFM. The exocytic frequency of TfnR-EGFP-containing vesicles was extremely decreased in cells treated with VAMP2 siRNAs (∼5 exocytic events/cell/min) (Fig. 7A,D, supplementary material Video S5), compared with the control cells (∼15 exocytic events/cell/min). On the other hand, the exocytic events of TfnR-EGFP-containing vesicles were not significantly affected in cells knocked down of VAMP3 or VAMP8 (Fig. 7C,D, supplementary material Videos S6 and S7, respectively). As shown in Fig. 7E, the decrease in the exocytic frequency of TfnR-EGFP-containing vesicles in VAMP2-knockdown cells was restored by exogenous expression of mCherry-tagged wild-type VAMP2, but not its mutant lacking the transmembrane domain (VAMP2ΔTM). Furthermore, the decrease in the exocytic frequency was not restored by exogenous expression of mCherry-VAMP3 or -VAMP8 (Fig. 7F). These observations together indicate that, among R-SNAREs, VAMP2 is primarily responsible for fusion of recycling vesicles containing Tfn–TfnR with the PM. This is in line with a recent observation that tRFP-tagged Sec8 and VAMP2-GFP are colocalized on the same peripheral vesicles undergoing exocytosis (Rivera-Molina and Toomre, 2013).

## DISCUSSION

**Fig. 8.**
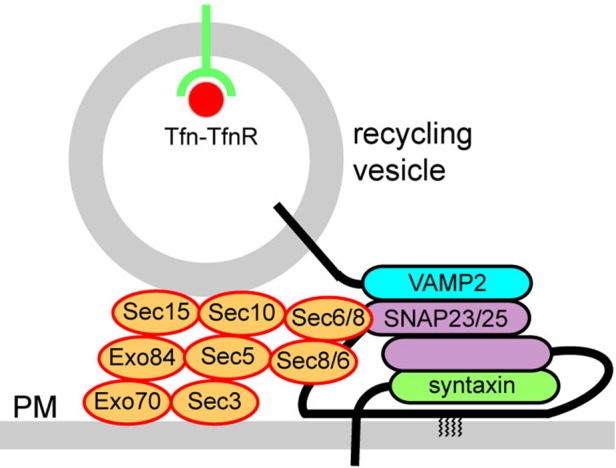
**A model for tethering and fusion of Tfn–TfnR-containing recycling vesicles with the PM, mediated by Rab11, the exocyst and SNAREs.** Rab11-positive recycling vesicles containing Tfn–TfnR are delivered to the cell periphery along microtubules. Rab11 on recycling vesicles interacts with Sec15, which in turn interacts with other exocyst subunits including Exo70. Exo70 probably binds to PtdIns(4,5)P_2_, thereby targeting the entire exocyst complex to the PM, while Sec6 and Sec8 interact with SNAP23/25 on the PM. By forming a *trans*-SNARE complex with VAMP2 on vesicles and an unknown syntaxin on the PM, SNAP23/25 mediates fusion of Tfn–TfnR-containing recycling vesicles with the PM.

Although Tfn and TfnR have been used as markers to trace the endocytic and recycling processes in a number of studies, regulation of their recycling process, particularly the final exocytic event, has been poorly characterized. On the basis of the data presented in this study, we propose a model for exocytosis of recycling vesicles containing Tfn–TfnR mediated by the exocyst and SNAREs (Fig. 8). In view of our previous study, recycling vesicles containing Tfn–TfnR are delivered to the cell periphery along microtubules (Takahashi et al., 2012). Since Rab11 interacts with Sec15 (Wu et al., 2005; Zhang et al., 2004) and Sec15 interacts with other exocyst subunits including Exo70 (Matern et al., 2001), it is likely that the exocyst is recruited onto Tfn–TfnR-containing recycling vesicles in a Rab11-dependent manner. On the other hand, the Exo70 subunit binds to PtdIns(4,5)P_2_, thereby targeting the entire exocyst complex to the PM (Liu et al., 2007). Our data presented in this study suggest that Q_bc_-SNAREs, SNAP23 and SNAP25 can interact with the exocyst complex through Sec6 and Sec8, respectively, thereby connecting vesicle tethering and fusion machineries. We further showed that knockdown of SNAP23 and/or SNAP25 leads to accumulation of Tfn and TfnR at the cell periphery and suppresses fusion of Tfn–TfnR-containing recycling vesicles with the PM; these phenotypes resemble those exhibited by Rab11-, Exo70- or Sec15-knockdown cells shown in our previous study (Takahashi et al., 2012). The involvement of SNAP23/25 in fusion of Tfn–TfnR-containing vesicles with the PM is in line with an early study showing that, in permeabilized MDCK cells, Tfn recycling is sensitive to botulinum neurotoxin E (Leung et al., 1998), which proteolytically cleaves SNAP23 and SNAP25.

Although VAMP2 was reported to be predominantly expressed in neuronal tissues (Elferink et al., 1989), it is expressed in 3T3-L1 adipocytes (Sadler et al., 2015; Williams and Pessin, 2008) and has been shown to be involved in regulated exocytosis of GLUT4-containing vesicles in response to insulin (Sadler et al., 2015; Sevilla et al., 1997). We showed that VAMP2 is expressed in non-neuronal cell lines, including HeLa, and primarily involved in fusion of Tfn–TfnR-containing vesicles with the PM in HeLa cells, although its expression level relative to the VAMP3 level is currently unknown. Our result is compatible with a recent observation that VAMP2-GFP are colocalized with Sec8-tRFP on peripheral vesicles that undergo exocytosis (Rivera-Molina and Toomre, 2013). By contrast, we failed to detect effects of VAMP3 knockdown on Tfn–TfnR recycling, although the VAMP3 protein was efficiently depleted. An early study of Galli et al*.* indicated an important role of VAMP3 in Tfn recycling, based on the observations that treatment of permeabilized CHO cells with TeTx impaired Tfn recycling, which correlated with proteolytic cleavage of VAMP3 mediated by the toxin (Galli et al., 1994). The same study, however, suggested that involvement of VAMP2 in Tfn recycling was unlikely although it is also sensitive to TeTx (Yamasaki et al., 1994), since immunoblot analysis failed to detect a significant level of VAMP2 in CHO cells. However, our data indicate that VAMP2 is a primary R-SNARE involved in the exocytic event of Tfn–TfnR-containing vesicles at least in HeLa cells, because we confirmed expression of the VAMP2 protein in HeLa cells and the VAMP2 transcript in non-neuronal cell lines, including HeLa, and because the decrease in the exocytic frequency of TfnR-EGFP-containing vesicles in VAMP2-knockdown cells can be restored by exogenous expression of VAMP2 but not by VAMP3. A previous immunoblot analysis using antibody recognizing multiple VAMPs detected a band corresponding to VAMP2 in addition to a VAMP3 band in a rat liver coated vesicle fraction (McMahon et al., 1993), supporting our data on expression of VAMP2 in non-neuronal cells. Thus, in this study, we showed for the first time that VAMP2 is involved in non-neuronal and non-regulated exocytosis.

Taken together, SNAP23/25 and VAMP2 are likely to form a *trans*-SNARE complex and mediate fusion of Tfn–TfnR-containing vesicles with the PM, downstream of Rab11 and the exocyst. Our data also provide novel insight into non-neuronal roles of VAMP2 and SNAP25, although these SNAREs are mainly expressed in neuronal tissues. On the other hand, our attempts to determine which Q_a_-SNARE(s) participates in fusion of recycling vesicles have so far been unsuccessful, since SNAP23/25 and VAMP2 can form complexes with various syntaxins, including syntaxin 2, syntaxin 3 and syntaxin 4 (Hong, 2005; Hong and Lev, 2014). It should be therefore addressed in future studies which Q_a_-SNARE(s) is involved in exocytosis of recycling vesicles, as well as whether VAMP2 is involved in vesicle fusion in other non-neuronal cells.

A previous study on recycling α5β1-integrin in macrophages showed that knockdown of VAMP3 or SNAP23 reduced cell surface expression of α5β1-integrin, although to a moderate extent (Veale et al., 2010). On the other hand, a previous systematic siRNA screening study using a HeLa cell line stably expressing an artificial secretory construct suggested that SNAP29 and syntaxin 19 could be involved in constitutive secretion of the artificial construct (Gordon et al., 2010); SNAP29, also known as GS32, is a remote homolog of SNAP23 and SNAP25, and associated with intracellular membranes, most enriched on Golgi membranes, and with the PM (Steegmaier et al., 1998; Wong et al., 1999), while very little is known about syntaxin 19. The same siRNA study did not detect requirement for SNAP23/25 or any of R-SNAREs in the constitutive secretion (Gordon et al., 2010). Taken together with our study, it is likely that fusion with the PM of recycling vesicles and that of constitutive secretory vesicles are mediated by distinct sets of SNARE proteins. On the other hand, cell surface expression of vesicular stomatitis virus G (VSVG) protein, a well-studied marker for the constitutive secretory pathway, was reported to be significantly retarded by knockdown of Exo70 (Liu et al., 2007), suggesting that the exocyst is required for constitutive exocytosis of VSVG at the PM. It is therefore an issue to be addressed in future studies how different kinds of vesicles have proper command of distinct tethering and/or fusion machineries.

## MATERIALS AND METHODS

### Antibodies and reagents

Monoclonal mouse anti-Exo70 antibody (70X13F3) was a kind gift from Shu-Chan Hsu (Rutgers University) (Vega and Hsu, 2001). Monoclonal mouse anti-TfnR antibody (H68.4) was purchased from Zymed. Polyclonal rabbit antibody that recognizes VAMP2 and VAMP3 (see Fig. 5A,B) was from Enzo Life Sciences. Polyclonal rabbit anti-VAMP8 was from Synaptic Systems. Monoclonal rabbit anti-SNAP25 antibody was from Abcam. Polyclonal rabbit anti-SNAP23 antibody and monoclonal mouse anti-TfnR antibody (CD71) were from Sigma-Aldrich. Monoclonal mouse antibodies against β-tubulin (KMX-1) and actin (C4) were from Millipore. Monoclonal mouse anti-Sec8 (clone 14) and anti-GFP (JL-8) antibodies were from BD Biosciences. Polyclonal rabbit anti-tagRFP antibody was from Evrogen. Polyclonal rabbit anti-RFP antibody that recognizes mCherry was from MBL. AlexaFluor-conjugated secondary antibodies and AlexaFluor 555-conjugated Tfn were from Molecular Probes. A DNA fragment for anti-GFP Nanobody synthesized based on the sequence used by Kubala et al. (2010) was subcloned into pGEX-6P-1 (GE Healthcare). The resulting plasmid was transformed into *Escherichia coli* BL21(DE3) cells, which were used for expression and purification of GST-tagged anti-GFP Nanobody.

### Plasmids

An expression vector for VAMP2, VAMP3, VAMP4, VAMP7 or VAMP8 was constructed by subcloning a cDNA fragment containing the entire coding sequence of corresponding human VAMP (see supplementary material Table S1) into pcDNA3-HAN (Shin et al., 1997). Expression vectors for the C-terminally truncated SNAP23 (ΔC9) and VAMP2 (ΔTM) was constructed by subcloning of cDNA fragments for the polypeptide region of SNAP23 and VAMP2, respectively (shown in supplementary material Table S1) into pcDNA3.1-mCherryC (a kind gift from Roger Tsien, UCSD) (Shaner et al., 2005). For co-immunoprecipitation experiments, the human SNAP23 or SNAP25 cDNA was subcloned into pEGFP-C1, and the cDNA for each human exocyst subunit was into pTagRFP-T-C1, pTagRFP-T-N1, (kind gifts from Hideki Shibata, Nagoya University) (Shibata et al., 2010), pcDNA3.1-mCherryC, pCAGneo-mCherryC or pCAGneo-mCherryN (Sakurai et al., 2012) as indicated. For siRNA rescue experiments, the SNAP23, VAMP2, VAMP3 or VAMP8 cDNA fragment was subcloned into pcDNA3.1-mCherryC.

### Co-immunoprecipitation analyses

Interactions between SNAP23/25 and exocyst subunits were examined using a procedure that will be described elsewhere in detail (Katoh et al., 2015). Briefly, HEK293T cells were transfected with EGFP and tRFP/mCherry fusion constructs for SNAP23/25 and an exocyst subunit, respectively, using Polyethylenimine Max (Polysciences), and cultured for 24 h. The cells were lysed in lysis buffer (50 mM Tris-HCl, pH 7.4, 150 mM NaCl and 0.1% NP-40) containing a protease inhibitor cocktail (Nacalai Tesque), and centrifuged for 15 min at 4°C in a microcentrifuge to remove cell debris. The supernatants were incubated with GST–anti-GFP-Nanobody pre-bound to glutathione–Sepharose 4B beads (GE Healthcare) at 4°C for 1 h. After washing the beads three times with lysis buffer, the materials bound to the beads were processed for immunoblot analysis using antibody against GFP, tRFP or RFP. For analysis of interactions of SNAP23/25 with endogenous exocyst subunits, HEK293T cells were transfected with an expression vector for EGFP-SNAP23 or -SNAP25, and lysates prepared from the transfected cells were subjected to immunoblot analysis using antibody against Sec8 or Exo70.

### DNA transfection and RNA interference

Plasmid DNAs were transfected into HeLa cells using X-tremeGENE 9 DNA Transfection Reagents according to the manufacturer's instructions (Roche Applied Science), For knockdown of the SNARE protein, a pool of siRNAs for each SNARE was prepared using the cDNA region shown in supplementary material Table S2 as a template with a BLOCK-iT RNAi TOPO transcription kit (Invitrogen) and a PowerCut Dicer kit (Thermo Scientific). Knockdown was performed as described previously (Takahashi et al., 2011). Briefly, HeLa cells were transfected with the siRNA pool using Lipofectamine 2000 (Invitrogen) and incubated for 24 h. The cells were then subcultured for 24 h, re-transfected with siRNAs and incubated for further 24 h. The cells were then subcultured, incubated for 48 h, and subjected to following analyses.

### Tfn recycling experiments, immunofluorescence microscopy and TIRFM

For Tfn recycling experiments, cells were serum-starved for 3 h, incubated with AlexaFluor 555-conjugated Tfn at 4°C for 50 min, and, after washing out excess fluorescent Tfn, incubated in serum-containing medium at 37°C for indicated time periods (Takahashi et al., 2012). Immunofluorescence analysis was performed as described previously (Takahashi et al., 2012, 2011). Briefly, cells were fixed with 3% paraformaldehyde at room temperature for 10 min, washed three times with phosphate-buffered saline (PBS), quenched with 50 mM NH_4_Cl for 20 min, washed three times with PBS, permeabilized with 0.1% TritonX-100 in PBS for 5 min, and washed three times with PBS. The fixed/permeabilized cells were then subjected to staining with primary and AlexaFluor-conjugated secondary antibodies, and observed using an Axiovert 200M microscope (Carl Zeiss) or an A1R-MP confocal laser-scanning microscope (Nikon).

TIRFM of HeLa cells stably expressing TfnR-EGFP (Takatsu et al., 2013) was performed as described previously (Takahashi et al., 2012). Briefly, the cells were treated with siRNAs as descried above and finally transferred to a glass-bottom culture dish. After 48 h incubation, the cells were incubated in HEPES-buffered modified Eagle's medium, placed on a microscope stage pre-warmed to 37°C, and observed at a video rate using NIS-Elements imaging software on a TIRFM ECLIPSE Ti (Nikon). Frames were taken every 90 ms.

### Statistical analysis

Statistical significance was evaluated by ANOVA with a Tukey-Kramer test.

## Supplementary Material

Supplementary Material
