# Supplementary material for: SNAP23/25 and VAMP2 mediate exocytic event of transferrin receptor-containing recycling vesicles
Source: Biol Open. 2015 Jun 19;4(7):910–20. doi: 10.1242/bio.012146 (PMC4571095; doi:10.1242/bio.012146)
Supplement: Supplementary Material [file supp_4_7_910__index.html]

SNAP23/25 and VAMP2 mediate exocytic event of transferrin receptor-containing recycling vesicles — Supplementary Material 

# SNAP23/25 and VAMP2 mediate exocytic event of transferrin receptor-containing recycling vesicles

## BIO012146 Supplementary Material

- Supplementary Material
