# Supplementary material for: SNAP23/25 and VAMP2 mediate exocytic event of transferrin receptor-containing recycling vesicles
Source: Biol Open. 2015 Jun 19;4(7):910–20. doi: 10.1242/bio.012146 (PMC4571095; doi:10.1242/bio.012146)
Supplement: Supplementary Material [file supp_bio.012146_BIO012146supp.pdf]

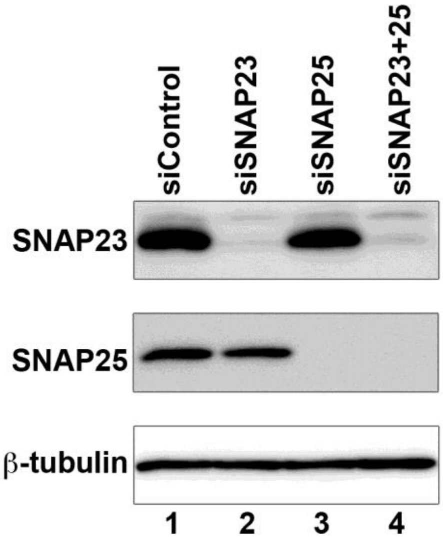

Fig. S1. Confirmation of knockdown of SNAP23 and/or SNAP25. HeLa cells were transfected with control siRNAs (siRNAs for LacZ, lane 1), or siRNAs for SNAP23 (lane 2) or SNAP25 (lane 3), or for both SNAP23 and SNAP25 (lane 4), and lysates prepared from the cells were subjected to immunoblot analysis for SNAP23 (top panel), SNAP25 (middle panel) or β-tubulin (bottom panel).

Table S1. Polypeptide regions of human VAMP and SNAP constructs used in this study

SNAREs	covered polypeptide regions
VAMP2	a.a. 1–116
VAMP2ΔTM	a.a. 1–94
VAMP3	a.a. 1–100
VAMP4	a.a. 1–141
VAMP7	a.a. 1–220
VAMP8	a.a. 1–100
SNAP23	a.a. 1–211
SNAP23ΔC9	a.a. 1–202
SNAP25	a.a. 1–206

Table S2. cDNA regions used for preparation of siRNA pools for human VAMPs and SNAPs. The A residue of the initiation Met codon of each cDNA is assigned as nucleotide residue 1. Note that the cDNA fragments of all, except for VAMP8, of the SNAREs cover only the 3'-untranslated regions.

SNAREs	cDNA regions
VAMP2	nucleotides 395–921
VAMP3	nucleotides 1,052–1,614
VAMP8	nucleotides 26–613
SNAP23	nucleotides 664–1,219
SNAP25	nucleotides 749–1,302

Supplementary Videos

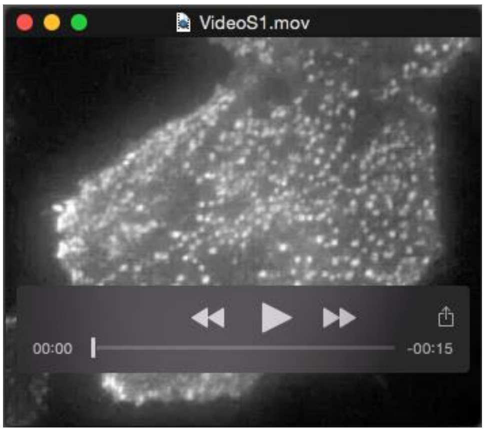

Video S1. TIRFM of cells expressing TfnR-EGFP. HeLa cells expressing TfnR-EGFP were treated with control siRNAs (siRNAs for LacZ) and subjected to TIRFM. Positions of typical exocytic events are circled in red.

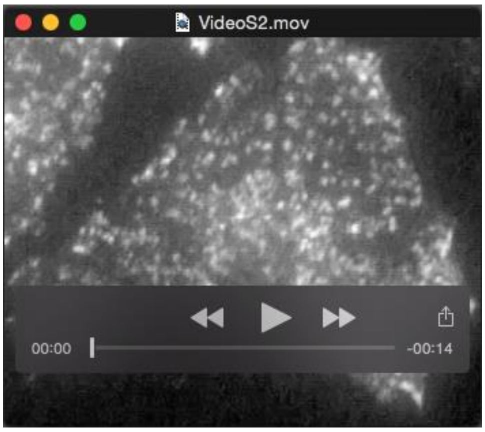

Video S2. TIRFM of cells expressing TfnR-EGFP and treated with SNAP23 siRNAs. HeLa cells expressing TfnR-EGFP were treated with SNAP23 siRNAs and subjected to TIRFM.

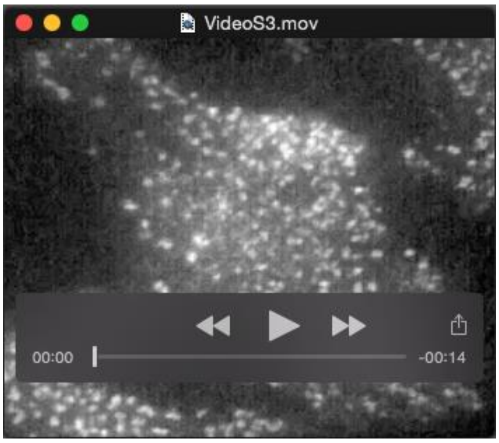

Video S3. TIRFM of cells expressing TfnR-EGFP and treated with SNAP25 siRNAs. HeLa cells expressing TfnR-EGFP were treated with SNAP25 siRNAs and subjected to TIRFM.

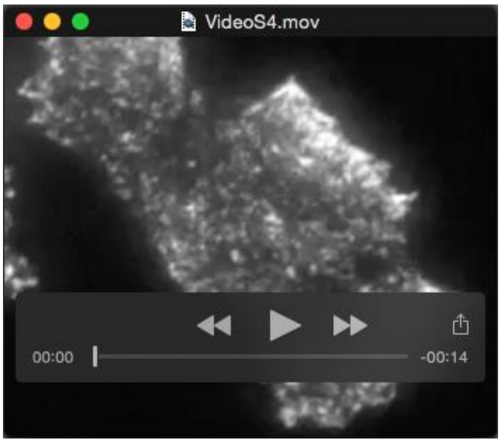

Video S4. TIRFM of cells expressing TfnR-EGFP and treated with SNAP23+SNAP25 siRNAs. HeLa cells expressing TfnR-EGFP were treated with siRNAs against SNAP23 and SNAP25, and subjected to TIRFM.

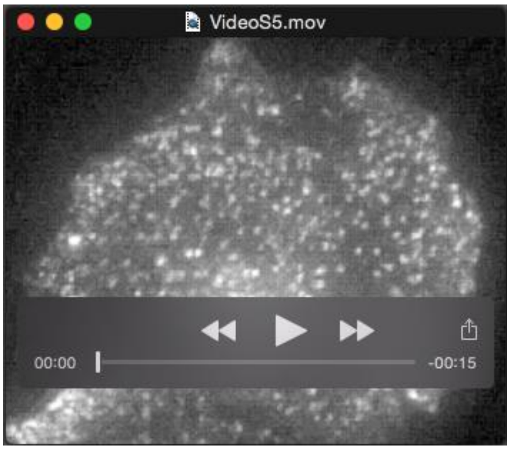

Video S5. TIRFM of cells expressing TfnR-EGFP and treated with VAMP2 siRNAs. HeLa cells expressing TfnR-EGFP were treated with VAMP2 siRNAs and subjected to TIRFM.

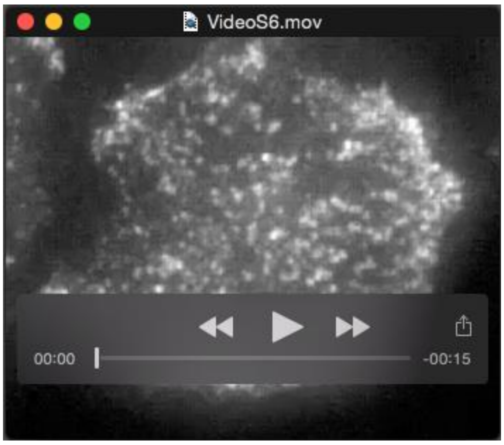

Video S6. TIRFM of cells expressing TfnR-EGFP and treated with VAMP3 siRNAs. HeLa cells expressing TfnR-EGFP were treated with VAMP3 siRNAs and subjected to TIRFM.

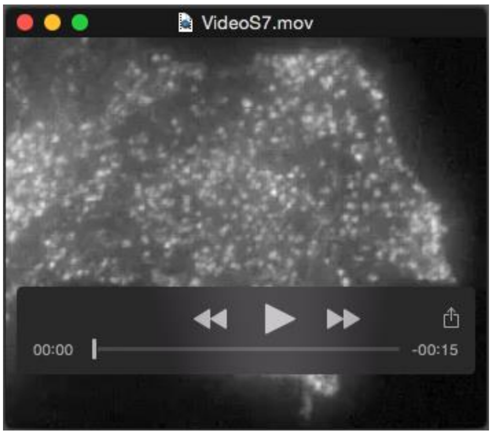

Video S7. TIRFM of cells expressing TfnR-EGFP and treated with VAMP8 siRNAs. HeLa cells expressing TfnR-EGFP were treated with VAMP8 siRNAs and subjected to TIRFM.
